# Transcriptome Analysis of the Cerebellum of Mice Fed a Manganese-Deficient Diet

**DOI:** 10.3389/fgene.2020.558725

**Published:** 2020-12-03

**Authors:** Young Ah Seo, Eun-Kyung Choi, Luisa Aring, Molly Paschall, Shigeki Iwase

**Affiliations:** ^1^Department of Nutritional Sciences, University of Michigan School of Public Health, Ann Arbor, MI, United States; ^2^Department of Human Genetics, Michigan Medicine, University of Michigan, Ann Arbor, MI, United States

**Keywords:** manganese, cerebellum, transcriptome, neurodevelopment, spliceosome

## Abstract

Manganese (Mn), primarily acquired through diet, is required for brain function and development. Epidemiological studies have found an association between both low and high levels of Mn and impaired neurodevelopment in children. Recent genetic studies have revealed that patients with congenital Mn deficiency display severe psychomotor disability and cerebral and cerebellar atrophy. Although the impact of Mn on gene expression is beginning to be appreciated, Mn-dependent gene expression remains to be explored in vertebrate animals. The goal of this study was to use a mouse model to define the impact of a low-Mn diet on brain metal levels and gene expression. We interrogated gene expression changes in the Mn-deficient mouse brain at the genome-wide scale by RNA-seq analysis of the cerebellum of mice fed low or normal Mn diets. A total of 137 genes were differentially expressed in Mn-deficient cerebellums compared with Mn-adequate cerebellums (*P*adj < 0.05). Mn-deficient mice displayed downregulation of key pathways involved with “focal adhesion,” “neuroactive ligand-receptor interaction,” and “cytokine-cytokine receptor interaction” and upregulation of “herpes simplex virus 1 infection,” “spliceosome,” and “FoxO signaling pathway.” Reactome pathway analysis identified upregulation of the splicing-related pathways and transcription-related pathways, as well as downregulation of “metabolism of carbohydrate,” and “extracellular matrix organization,” and “fatty acid metabolism” reactomes. The recurrent identifications of splicing-related pathways suggest that Mn deficiency leads to upregulation of splicing machineries and downregulation of diverse biological pathways.

## Introduction

The micronutrient manganese (Mn) plays important roles in fundamental cell functions and various physiological processes, such as protein glycosylation, detoxification of superoxide, bone formation, immune responses, and carbohydrate metabolism (Horning et al., 2015). The critical roles of Mn in these processes are due to its participation as a cofactor for numerous enzymes, including arginase, xanthine oxidase, galactosyltransferase, pyruvate decarboxylase, glutamine synthetase, and Mn superoxide dismutase (Horning et al., 2015). The human body contains ∼10–20 mg Mn, with the highest concentrations found in bone, liver, pancreas, kidney, and brain tissues (Schroeder et al., 1966; Aschner and Aschner, 2005). Diet is a major source of Mn intake in humans, and ∼1–5% of the ingested Mn is absorbed (Davis et al., 1993). The primary source of dietary Mn is mostly plant-based foods, such as whole grains, legumes, rice, nuts, and vegetables, whereas this nutrient is relatively deficient in animal sources (Freeland-Graves et al., 2016).

Manganese is required for the functioning and development of the brain, one of the most metabolically active organs in the body. Mn levels in the brain increase after birth, and the incorporation of Mn into the brain is most remarkable in young rats (Markesbery et al., 1995), suggesting that this essential metal plays a pivotal role in brain development (Zoni and Lucchini, 2013). Mn deficiency is associated with epilepsy in humans and rats (Carl et al., 1993). Epidemiological studies have shown that low and high Mn levels are associated with impaired neurodevelopment (Claus Henn et al., 2010; Bhang et al., 2013; Haynes et al., 2015). Recent genetic studies have revealed that patients with congenital Mn deficiency display severe psychomotor disability, cerebral and cerebellar atrophy, seizures, and vision and hearing impairment (Boycott et al., 2015; Park et al., 2015; Riley et al., 2017). In addition to its developmental roles, Mn has been associated with motor function in adults. For example, exposure to high levels of Mn in occupational settings (e.g., welding, mining, dry battery manufacturing, etc.) can lead to Mn accumulation in the brain and a Parkinsonian-like disorder known as manganism (Olanow, 2004; Perl and Olanow, 2007). Overall, most studies have associated altered Mn levels, both excessive and insufficient, with altered brain function in humans and model organisms.

Despite the critical roles of Mn in the brain, the underlying mechanisms of Mn-dependent structural and functional changes in the central nervous system (CNS) remain incompletely characterized. Therefore, understanding the impact of Mn on CNS gene regulation is an essential step in elucidating the mechanisms underlying Mn-dependent brain dysfunction. Previous RNA-seq analysis in *Caenorhabditis elegans* showed that Mn exposure increases pathways related to endoplasmic reticulum and lipocalin (Rudgalvyte et al., 2016). Another transcriptomic analysis in human SH-SY5Y neuroblastoma cells has revealed distinct responses to physiologic and toxic Mn exposure (Fernandes et al., 2019). The impact of Mn on gene expression is beginning to be appreciated, but Mn-dependent gene expression remains poorly explored in vertebrate animals. Given that Mn is primarily acquired through diet, the goal of the present study was to define the impact of low levels of dietary Mn on brain metal levels and to probe the molecular dysfunction associated with low Mn levels in an unbiased manner using a mouse model.

## Materials and Methods

### Animals and Treatment Conditions

Four-week-old male wild-type (WT) C57BL/6 mice were purchased from Jackson Laboratory and maintained on a metal-basal diet containing 35 ppm Mn (TD120518, Harlan Teklad, Indianapolis, IN, United States; Supplementary Table 1), as previously described (Seo and Wessling-Resnick, 2015; Seo et al., 2016; Choi et al., 2020). The trace element levels in the diet were as recommended by the American Institute of Nutrition (Reeves et al., 1993). For dietary Mn alteration, the mice were fed either low-Mn or normal-Mn diets (< 0.01 or 35 ppm Mn, respectively; Harlan Teklad). The actual Mn concentrations of the diets were 0–0.5 ppm Mn (low-Mn) and 35–35.5 ppm Mn (normal-Mn), as determined by inductively coupled plasma mass spectrometry (ICP-MS; Choi et al., 2018, 2020). This study was performed in strict accordance with the recommendations in the Guide for the Care and Use of Laboratory Animals of the National Institutes of Health (Bethesda, MD, United States). The protocol (protocol number: PRO00008963) was approved by the University Committee on Use and Care of Animals (UCUCA) at the University of Michigan.

### RNA Isolation and Sequencing

Total RNA was isolated from one cerebellum per mouse using the RNeasy mini kit (Qiagen, Valencia, CA, United States). Three mice (*n* = 3 biological replicates) were used per experimental group (total *n* = 6). The sample size was determined based on previously described power calculations to optimize detection of differentially expressed genes (Ching et al., 2014). RNA concentrations were measured with an Epoch Microplate Spectrophotometer (BioTek, Winooski, VT, United States). RNA integrity was assessed with an Agilent Bioanalyzer 2100 using a Nano 6000 assay kit (Agilent Technologies, Santa Clara, CA, United States). An RNA integrity number (RIN) > 7.2 was considered the minimum requirement for library preparation. RNA was reverse transcribed into cDNA using oligo-dT, and cDNA libraries were generated with a NEBNext Ultra II RNA Library Prep Kit (NEB #E7775). An insert size of 250–300 bp was used for cDNA library preparation. Libraries were sequenced on the Illumina NovaSeq 6000 platform with a 150-bp paired-end mode. Reference genome and annotation files were downloaded from Ensembl, and RNA-seq data were aligned to the reference genome using the spliced transcripts alignment to a reference (STAR) software (Dobin et al., 2013). The DESeq2 package was used for differential expression analysis. The ClusterProfiler, ReactomePA, and KEGG databases were used for enrichment pathway analysis (Ogata et al., 1999).

### Quantitative PCR (qPCR)

Purified RNA (2 μg) was reverse-transcribed with SuperScript III First-Strand Synthesis System (Invitrogen; Thermo Fisher Scientific, Inc.). qPCR reactions were carried out using the Power SYBR Green PCR master mix containing 0.5 μM of forward and reverse primers and 2% of cDNA generated from 2 μg of RNA. The conditions were 95°C for 10 min, followed by 45 cycles of 95°C for 15 s and 60°C for 2 min. Specific primers were designed using Primer 3 software (Untergasser et al., 2012). The efficiency of the primers used for qPCR was validated by creating standard curves using a gradient of diluted samples with *r*^2^ values larger than 0.96. The specificity of the primers was verified by examining the melting curve. 36B4 was used for normalization of the mRNA because the 36B4 gene is highly conserved and encodes acidic ribosomal phosphoprotein P0 (RPLP0) that is a component of the 60S ribosomal subunit (Akamine et al., 2007). The relative gene transcription levels were calculated using the 2^–Δ^ ^Δ^ ^*Ct*^ method (Pfaffl, 2001). The primers used for qPCR are listed in Supplementary Table 2 and were all purchased from Integrated Genomics Technologies.

### Trace Element Analysis

Brain metal levels were measured by ICP-MS, as previously described (Seo and Wessling-Resnick, 2015; Seo et al., 2016; Choi et al., 2020). Briefly, brain samples taken from the mice were digested with 2 mL/g total wet weight nitric acid (BDH ARISTAR^®^ ULTRA) for 24 h and then digested with 1 mL/g total wet weight hydrogen peroxide (BDH^®^ Aristar ULTRA) for 24 h at room temperature. Specimens were preserved at 4°C until quantification of metals. Ultrapure water was used for final sample dilution.

### Statistical Analysis

Results are presented as means ± SEM. Statistical comparisons were determined with Student’s *t*-test using Prism 7 software (GraphPad Software). Values of *P* < 0.05 were considered statistically significant. Asterisks in graphs, wherever present, denote statistically significant differences.

## Results

### Dietary Mn Alters Brain Mn Concentrations in Mice

**FIGURE 1:**
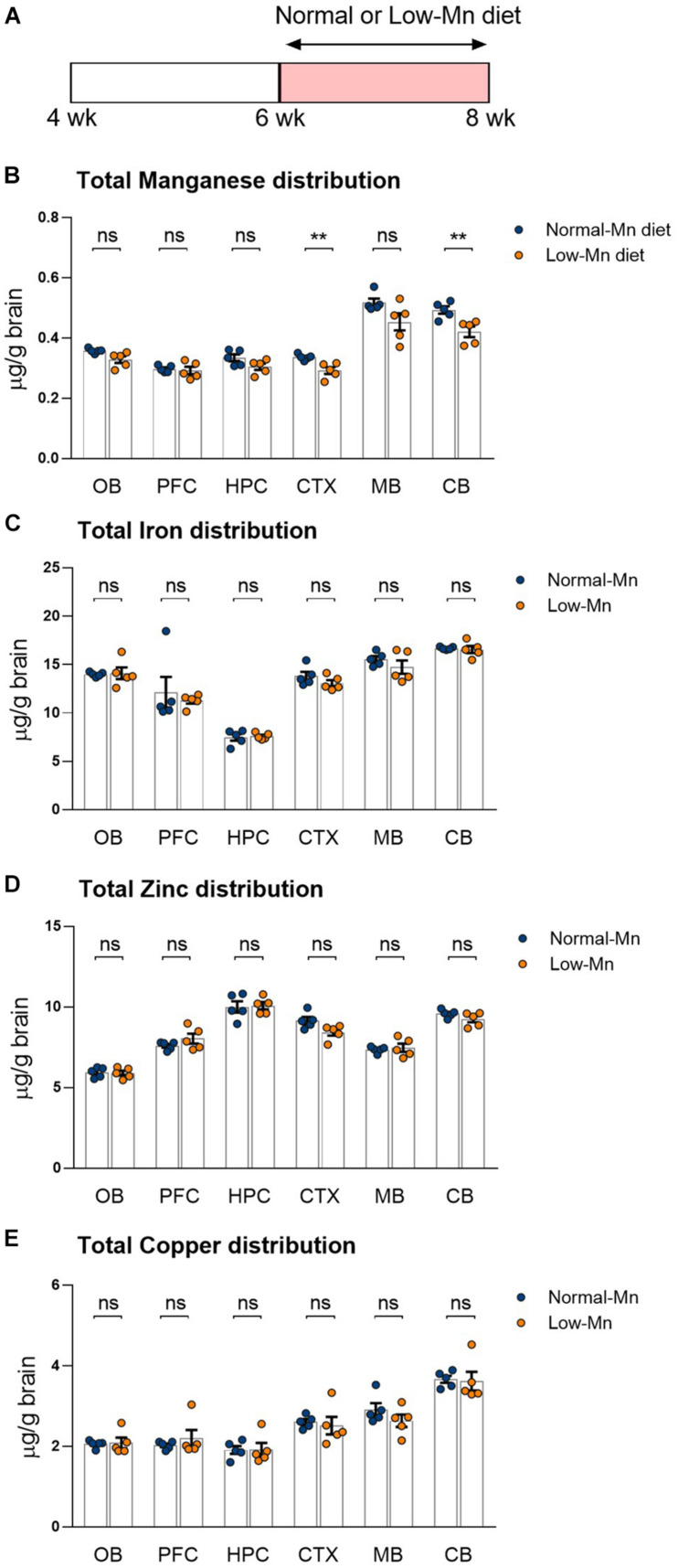
Dietary manganese (Mn) depletion reduces brain Mn levels. **(A)** Experimental scheme to analyze the transcriptome in low-Mn or normal-Mn diets fed mice. Mice were fed with either low-Mn or normal-Mn diets (0 and 35 mg/kg Mn, respectively). **(B–E)** Levels of the trace elements Mn, iron (Fe), zinc (Zn), and copper (Cu) were measured by inductively coupled plasma mass spectrometry (ICP-MS) in the brain regions of mice (*n* = 5 per group). Data represent means ± SEM. ** *P* < 0.01.

We determined the effect of low levels of dietary Mn on the brain metal levels by measuring the distribution of metals in various regions of the brain by ICP-MS. Mice were fed for 14 days with purified diets that contained either low or normal concentrations of Mn (< 0.01 or 35 ppm Mn, respectively; Figure 1A). The mice fed with a low-Mn diet showed significantly reduced Mn levels in different brain regions, including the cortex (∼13.1%; *P* < 0.01) and cerebellum (∼14.7%; *P* < 0.01), when compared to the mice fed a normal-Mn diet (Figure 1B). The same samples showed no alterations in the levels of other trace elements, including iron, zinc, and copper, in the brain regions (Figures 1C–E). No heavy metals, such as cadmium and lead, were detected in these samples. No differences were found in body weight changes (3.74 vs. 3.40 g; *P* = 0.4356) and food intake (2.39 vs. 2.47 g/mouse/day; *P* = 0.7399) during the experimental period. These findings demonstrate that 2 weeks of low level of Mn reduce the Mn levels in the cortex and cerebellum without affecting other trace element levels.

### Transcriptome Analysis of Cerebellum in Mice Fed Diets Containing Low or Normal Mn Levels

**FIGURE 2:**
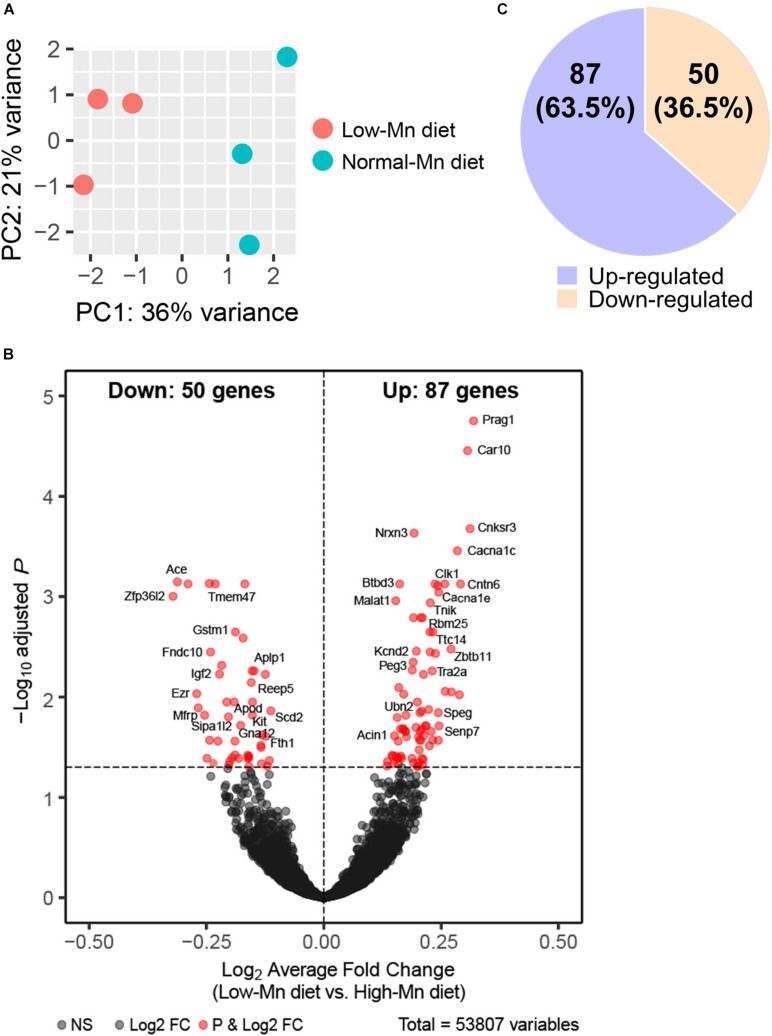
RNA-Seq analysis of manganese (Mn)-deficient cerebellum in mice. **(A)** Principal component analysis (PCA) plot showing the clustering of each of the samples with technical triplicates along two principle components (PC1 – 36% variance; PC2 – 21% variance). Each technical triplicate clusters with the other. **(B)** DESeq reveals 137 altered genes (significantly altered genes defined as a *P*adj-value < 0.05). Volcano plot profiles –log10 *P*adj-value and log2-fold change of gene expression between Mn-deficient vs. normal cerebellum samples. **(C)** 50 genes (36.5%) are downregulated and 87 genes (63.5%) are upregulated.

We interrogated gene expression changes in the Mn-deficient mouse brain at a genome-wide scale by RNA-seq analysis of the cerebellum from mice fed either low (0 ppm) or normal (35 ppm) Mn diets. The cerebellum was chosen over other brain regions for the following reasons. First, when compared with mice fed a normal Mn diet, mice fed a low-Mn diet showed the largest reduction in brain Mn levels in the cerebellum (Figure 1B). Second, human patients with congenital Mn deficiency display neurodevelopmental conditions associated with cerebellum atrophy (Boycott et al., 2015; Park et al., 2015; Riley et al., 2017). We harvested cerebellum tissues from three mice per group. We then prepared cDNA libraries of poly adenylated mRNAs and subjected them to high-throughput sequencing using the Illumina NovaSeq platform. At least 17 million uniquely mapped reads were obtained per sample. Principal component analysis (PCA) showed that the three replicates clearly cluster together and segregated into the two Mn treatment groups, indicating that Mn deficiency triggered transcriptomic alterations (Figure 2A).

We next sought to identify altered genes in the cerebellum of mice fed low or normal Mn diets. Differentially expressed (DE) genes were determined by DESeq2 (Love et al., 2014) for mice fed low or normal Mn diets. We found 137 genes (0.25% of 53807 total annotated genes) that were differentially regulated with an adjusted *p*-value (*P*adj) < 0.05 in the cerebellum of mice fed a low Mn diet (Supplementary Data 1). Of these, 87 genes were upregulated (63.5%), whereas 50 genes were downregulated (36.5%; Figures 2B,C). The magnitude of the changes was relatively moderate, as log_2_fold changes ranged from −0.40 to 0.43. The genes with the 20 largest log_2_fold changes (downregulated and upregulated) are presented in Tables 1, 2. Transcripts that decreased in the Mn-deficient cerebellum included genes related to anti-aging, CNS myelination, genome integrity, cell adhesion and migration processes, fatty acid metabolism, and postsynaptic function. The results also showed a number of genes that increased in the Mn-deficient cerebellum; these included genes involved in apoptosis suppression, circadian rhythms, regulation of neurite growth, and neurodevelopment. Taken together, these results indicate that Mn deficiency leads to misregulation of specific genes in the cerebellum.

**TABLE 1:** The 20 most downregulated genes in the manganese (Mn)-deficient mouse cerebellum.

Gene symbol	Gene description	log2 fold- change	*P*adj	Known function
**Down-regulated**				
Kl	Klotho	–0.40	5.36E-07	Anti-aging (Kim et al., 2015)
Plp1	Proteolipid protein 1	–0.22	3.38E-06	CNS myelination (Garbern et al., 2002)
H3f3b	H3.3 Histone B	–0.22	6.62E-06	Genome integrity (Jang et al., 2015)
Fn1	Fibronectin 1	–0.31	7.13E-04	Cell adhesion and migration processes
Vat1l	Vesicle amine transport 1 like	–0.24	7.39E-04	Unknown function
Ace	Angiotensin I converting enzyme	–0.29	7.47E-04	Converts angiotensin I to angiotensin II
Tmem47	Transmembrane protein 47	–0.23	7.47E-04	Localized in ER and plasma membrane; unknown function
Slc4a4	Sodium bicarbonate co-transporter	–0.17	7.47E-04	bicarbonate secretion and absorption (Burnham et al., 1997)
Zfp36l2	ZFP36 ring finger protein like 2	–0.32	9.93E-04	mRNA decay activator protein (Stumpo et al., 2009)
Gstm1	Glutathione S-transferase Mu 1	–0.19	2.25E-03	Detoxification of electrophilic compounds
Zbtb18	Zinc finger and BTB domain containing 18	–0.17	2.58E-03	Transcriptional repressor in myogenesis and brain development (Aoki et al., 1998)
Fndc10	Fibronectin type III domain containing 10	–0.24	3.56E-03	Unknown function
Hsd17b12	Hydroxysteroid 17-beta dehydrogenase 12	–0.22	4.83E-03	Fatty acid elongation (Moon and Horton, 2003)
Aplp1	Amyloid beta precursor like protein 1	–0.15	5.50E-03	AD-associated gene; postsynaptic function
Trf	Transferrin	–0.15	5.50E-03	Iron storage protein
Igf2	Insulin like growth factor 2	–0.22	5.91E-03	Major fetal growth hormone in mammals (Livingstone and Borai, 2014)
Eef1a1	Eukaryotic translation elongation factor 1 alpha 1	–0.12	5.95E-03	Recruitment of aminoacyl-tRNA to ribosome during protein synthesis (Maruyama et al., 2007)
Reep5	Receptor accessory protein 5	–0.15	7.18E-03	Signaling by GPCR and olfactory transduction
Ezr	Ezrin (villin 2)	–0.27	9.27E-03	Connections of cytoskeletal structures to the plasma membrane (D’Angelo et al., 2007)
Apod	Apolipoprotein D	–0.19	1.12E-02	A component of high-density lipoprotein; mainly produced in the brain and testes (Muffat and Walker, 2010)

**TABLE 2:** The 20 most upregulated genes in the manganese (Mn)-deficient mouse cerebellum.

Gene symbol	Gene description	log2 fold-change	*P*adj	Known function
**Up-regulated**				
Meg3	Maternally expressed 3	0.34	5.60E-27	long non-coding RNAs; apoptosis suppression (Chen et al., 2011)
Ddit4	DNA-damage-inducible transcript 4	0.42	5.78E-08	Negative regulator of mTOR (Sofer et al., 2005)
Per1	Period circadian regulator 1	0.36	5.78E-08	Maintenance of circadian rhythms in cells (Angelousi et al., 2018)
Tob2	Transducer of ERBB2, 2	0.39	3.79E-07	Anti-proliferative protein
Bcl6	B-cell lymphoma 6 protein	0.43	5.36E-07	Transcription repressor (Basso et al., 2010)
Slc38a2	Sodium-coupled neutral amino acid transporter 2	0.25	1.19E-06	Functions as a sodium-dependent amino acid transporter (Hatanaka et al., 2000)
Sgk1	Serum/glucocorticoid regulated kinase 1	0.39	1.50E-06	Cellular stress response (Lang and Shumilina, 2013)
Prag1	PEAK1 related, kinase-activating pseudokinase 1	0.32	1.77E-05	Regulation of neurite outgrowth (Tanaka et al., 2006)
Car10	Carbonic anhydrase 10	0.31	3.51E-05	Brain development (Sterky et al., 2017)
Cnksr3	Connector enhancer of kinase suppressor of Ras 3	0.31	2.10E-04	Involved in transepithelial sodium transport (Soundararajan et al., 2012)
Nrxn3	Neurexin-3-alpha	0.19	2.33E-04	CNS receptors and cell adhesion molecules (Muskiewicz et al., 2018)
Cacna1c	Calcium voltage-gated channel subunit alpha1 C	0.28	3.50E-04	Influx of Ca2 + into the cell upon membrane polarization (Shaw and Colecraft, 2013)
Clk1	CDC like kinase 1; dual specificity protein kinase	0.24	7.47E-04	Phosphorylation of serine- and arginine-rich proteins of the spliceosomal complex (Moeslein et al., 1999)
Zgpat	Zinc finger CCCH-type and G-patch domain containing	0.26	7.47E-04	Transcription repressor
Cntn6	Contactin 6	0.29	7.47E-04	Cell surface interactions during CNS development (Zuko et al., 2016)
Btbd3	BTB domain containing 3	0.16	7.47E-04	A key regulator of dendritic field orientation during development of sensory cortex (Matsui et al., 2013)
Rbm33	RNA binding motif protein 33	0.24	7.78E-04	unknown function
Cacna1e	Calcium voltage-gated channel subunit alpha1 S	0.24	9.05E-04	Entry of calcium ions into excitable cells (Helbig et al., 2019)
Malat1	Metastasis associated lung adenocarcinoma transcript 1	0.15	1.10E-03	A large, infrequently spliced non-coding RNA (Amodio et al., 2018)
Tnik	TRAF2 And NCK interacting kinase	0.23	1.15E-03	Activator of the Wnt signaling pathway (Mahmoudi et al., 2009)

### RNA-Seq Pathway Analysis in the Mn-Deficient Cerebellum

**FIGURE 3:**
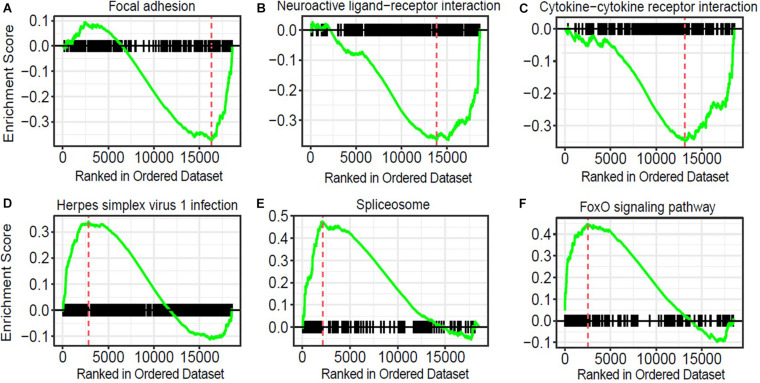
Pathway analysis of genes correlated with manganese (Mn)-deficient cerebellum. Gene sets and their associated enrichment plots generated by gene set enrichment analysis (GSEA) of pre-ranked gene expression data (*P*adj < 0.05). Enrichment plot with enrichment scores (ES) and differentially expressed genes are shown for **(A)** focal adhesion, **(B)** neuroactive ligand-receptor interaction, and **(C)** cytokine–cytokine receptor interaction, **(D)** herpes simplex virus 1 infection, **(E)** spliceosome, and **(F)** FoxO signaling pathway. The *X*-axis denotes the position of the pathway genes in all analyzed genes, which are ranked in an order based on fold changes (high to low) by Mn deficiency. The enrichment score is shown as a curve, and the vertical red bars in the plot indicate the position of the “leading edge” of the enrichment. For downregulated pathways **(A–C)**, the genes with higher rank orders than the red line, on the right side of the red line, contributed to the downregulation of the three pathways. Conversely, for the upregulated pathways **(D–F)**, the genes with lower rank orders, on the **left** side of the red line, contributed to their upregulations.

To obtain insights into the biological processes in the brain that are influenced by the relatively moderate changes in a number of genes, we applied gene set enrichment analysis (GSEA; Subramanian et al., 2005) to our RNA-Seq data. With the GSEA algorithm, we tested the enrichment of biological pathways annotated by the Kyoto Encyclopedia of Genes and Genomes (KEGG), a database resource integrating genomic, chemical, and systemic functional information (Kanehisa and Goto, 2000). We identified three downregulated and three upregulated pathways in the Mn-deficient cerebellum (*P*adj < 0.05; Figure 3). The downregulated pathways are “focal adhesion,” “neuroactive ligand-receptor interaction,” and “cytokine-cytokine receptor interaction,” while upregulated pathways include “herpes simplex virus 1 infection,” “spliceosome,” and ‘FoxO signaling pathway.” In Table 3, we list the top-ranked genes from the GSEA analysis that are also significantly altered in the DEseq2 analysis. Notably, upregulation of the spliceosome pathway is represented by the following six top-ranked, significantly altered genes: *Rbm25, Ddx5, Srsf5, Acin1, Tra2a*, and *Snrnp70*. Other KEGG pathways contain fewer highly altered genes, notably *Srsf5* for Herpes simplex virus 1 infection; *Sgk1, Bcl6*, and *Ccng2* for FoxO signaling; and *Fn1* and *Actg1* for the focal adhesion pathway (Table 3).

**TABLE 3:** Pathway analysis of genes correlated with the manganese (Mn)-deficient mouse cerebellum.

KEGG pathway (ID)	Gene symbol	Gene description	log2 fold-change	*P*adj	Known function
Spliceosome (mmu03040)	Rbm25	RNA binding motif protein 25	2.10E-01	1.62E-03	Alternative pre-mRNA splicing regulator; apoptosis (Fortes et al., 2007)
	Ddx5	DEAD-box helicase 5, p68	1.51E-01	2.43E-02	RNA helicase; interactions with other factors (Fuller-Pace and Ali, 2008)
	Srsf5	Serine and arginine rich splicing factor 5	1.49E-01	4.59E-02	Splicing regulator; an insulin-induced gene (Diamond et al., 1993)
	Acin1	Apoptotic chromatin condensation inducer 1	1.69E-01	2.08E-02	Apoptotic chromatin condensation after activation by caspase-3, without inducing DNA fragmentation (Sahara et al., 1999)
	Tra2a	Transformer 2 alpha homolog	2.31E-01	5.50E-03	Pre-mRNA splicing; Invasion and epithelial mesenchymal transition of glioma cells (Tan et al., 2018)
	Snrnp70	Small nuclear ribonucleoprotein U1 subunit 70	1.59E-01	4.02E-02	Mixed connective tissue disease and systemic scleroderma; Alzheimer’s disease brains-associated gene (Hales et al., 2014)
Herpes simplex virus 1 infection (mmu05168)	Srsf5	Serine and arginine rich splicing factor 5	1.49E-01	4.59E-02	A major regulator of human immunodeficiency virus type 1 mRNA splicing (Hallay et al., 2006)
FoxO signaling pathway (mmu04068)	Sgk1	Serum/glucocorticoid regulated kinase 1	3.94E-01	1.50E-06	Cellular stress response and neuronal function (Lang et al., 2010)
	Bcl6	BCL6 transcription repressor	4.30E-01	5.36E-07	Neuronal function; schizophrenia, depression, and Parkinson’s, and Alzheimer’s disease-associated gene (Basso et al., 2010)
	Ccng2	Cyclin G2	2.72E-01	8.90E-03	Eukaryotic cell cycle; cell proliferation as a tumor suppressor gene (Zhang et al., 2018)
Focal adhesion (mmu04510)	Fn1	Fibronectin 1	−3.12E-01	7.13E-04	Cell adhesion and migration processes including embryogenesis, wound healing, blood coagulation, host defense, and metastasis (Liao et al., 2018)
	Actg1	Actin gamma 1	−1.32E-01	2.39E-02	Cell motility and maintenance of the cytoskeleton; Gene mutations associated with sensorineural progressive hearing loss and with Baraitser-Winter syndrome (Verloes et al., 2015)

### Reactome Alterations in the Mn-Deficient Cerebellum

**FIGURE 4:**
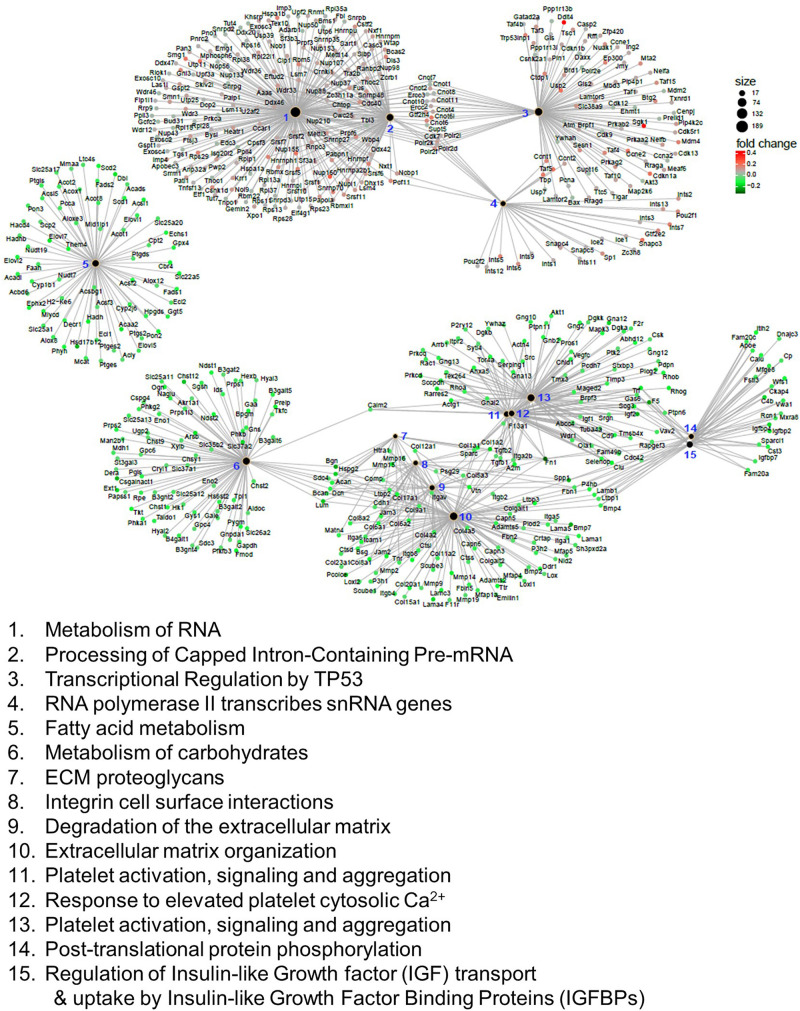
Reactome analysis of genes correlated with manganese (Mn)-deficient cerebellum. Enrichment of reactome categories, as calculated with the gene set enrichment analysis (GSEA) algorithm. Among the 62 reactomes (*P*adj < 0.05), the top 15 reactomes based on *P*adj values are represented. The size of the black dots indicates the number of genes involved in the reactome. Genes involved in the reactome are colored based on the mRNA changes in the Mn-deficient cerebellum. Reactomes can be interconnected via genes that belong to multiple reactomes.

We also applied GSEA analysis to test the enrichment of the reactome, which encompasses physical protein-protein interactions that may not be covered in KEGG (Yu and He, 2016). In this analysis, we identified 62 reactomes that are either upregulated or downregulated in the Mn-deficient cerebellum (Padj < 0.05). Again, we identified the upregulation of spliceosome-related reactomes, including “metabolism of RNA” and “processing of capped intron-containing pre-mRNA.” We visualized the top 15 reactomes and the genes contributing to the enrichment in a network representation (Figure 4). The network assembly revealed that upregulation of the splicing-related and transcription-related reactomes, including “transcriptional regulation of TP53” and “RNA polymerase II transcribes snRNA genes,” and the two modules were linked by genes that belonged to both splicing and transcription modules. Similarly, downregulated reactomes, such as “metabolism of carbohydrate,” and “extracellular matrix organization” were linked by a set of genes. By contrast, “fatty acid metabolism” was isolated from the other downregulated reactomes. Recurrent identifications of splicing-related pathways strongly suggested that Mn deficiency led to upregulation of the splicing machineries. At the same time, downregulation of diverse biological pathways could occur when Mn was insufficient.

### qPCR Analysis of the Cerebellum in Mice Fed a Low-Mn Diet for 2 or 4 Weeks

**FIGURE 5:**
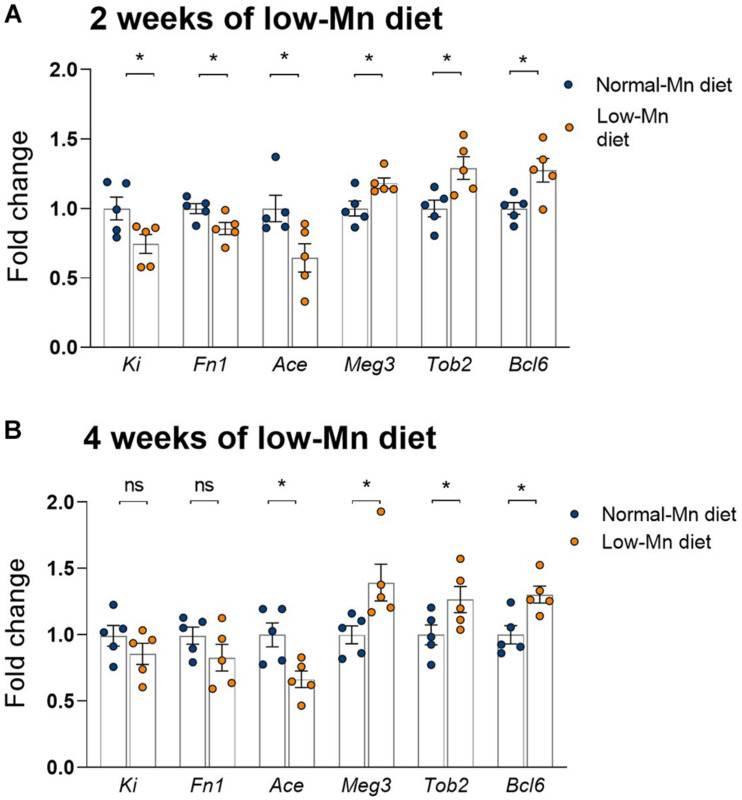
qPCR analysis of the cerebellum of mice fed for 2 or 4 weeks with a low-manganese (Mn) diet. **(A)** qPCR validation of *Ki*, *Fn1*, *Ace*, *Meg3*, *Tob2*, and *Bcl6*, six genes shown to be dysregulated in the RNA-seq analysis of the cerebellum of mice fed for 2 weeks with a low-Mn diet (*n* = 5 per group). **(B)** qPCR quantification of *Ki*, *Fn1*, *Ace*, *Meg3*, *Tob2*, and *Bcl6* in the cerebellum of mice fed for 4 weeks with a low-Mn diet (*n* = 5 per group). Data represent means ± SEM. *P < 0.05.

We used qPCR to validate several genes showing significant alterations in the cerebellum of mice fed a Mn-deficient diet for 2 weeks. We found that dietary Mn deficiency for 2 weeks led to the downregulation of *Ki*, *Fn1*, and *Ace* in the cerebellum. These three genes were also shown to be downregulated in the RNA-seq analysis (Figure 5A). We also found that three genes that were upregulated in the RNA-seq analysis, namely *Meg3*, *Tob2*, and *Bcl6*, showed similar changes in the qPCR analysis (Figure 5A). We then examined whether the effects of Mn deficiency would be more extreme if the mice were exposed to the low-Mn diet for longer than 2 weeks. To test this, we performed additional experiments in mice fed the low-Mn diet for 4 weeks. Our qPCR data revealed similar changes in mice fed the low-Mn diet for either 2 or 4 weeks (Figure 5B). This result indicated that an additional 2 weeks of a low-Mn diet did not exacerbate the Mn-related gene misregulation.

## Discussion

Our use of a mouse model of the Mn-deficient cerebellum shows that Mn deficiency results in altered expression of 137 genes within the adult cerebellum. The present results demonstrate upregulation of transcripts of the spliceosome-related pathways in the Mn-deficient mouse cerebellum. These genes include *Rbm25*, *Ddx5*, *Srsf5*, *Acin1*, *Tra2a*, and *Snrnp70*, all of which play central roles in the spliceosome pathway (Table 3). Most eukaryotic genes are transcribed to precursor mRNAs (pre-mRNAs) that encompass both protein-coding exons and non-coding introns. Pre-mRNA splicing removes the non-coding intron sequences to produce the mature mRNA. This sequential process is catalyzed by a macromolecular machine called the spliceosome (Zaghlool et al., 2014). The spliceosome plays critical roles in neurodevelopmental disorders, as splice-disrupting genetic variants are known to contribute to these disorders (Sanders et al., 2020). The mechanisms by which Mn deficiency alters splicing machinery remain unknown; however, our observations raise the intriguing possibility that Mn may contribute to neurodevelopmental disorders by altering gene splicing.

One example is RBM25 (also known as RED120), an RNA-binding protein that contains an N-terminal RNA-binding motif domain (RRM), a central glutamate/arginine-rich sequence (ER-rich domain), and a C-terminal PWI domain (Fortes et al., 2007). RBM25 functions in apoptotic cell death by regulating the balance of expression of pro- and anti-apoptotic transcripts of the BCL2L1 gene isoforms (Zhou et al., 2008). RBM25 also generates an abnormal and truncated splice form of the cardiac voltage-gated Na channel encoded by SCN5A during heart failure (Gao et al., 2011). Another example is DDX5, or DEAD/H box polypeptide 5, also known as RNA helicase p68, that is involved in the alternate regulation of pre-mRNA splicing. DDX5/RNA helicase p68 is reported to regulate tau exon 10 splicing by modulating the stem-loop structure at the 5′ splice site (Forman et al., 2004), and disruption of the regulation of tau exon 10 splicing plays an important role in the pathogenesis of tauopathy (Forman et al., 2004). Moreover, aberrant tau exon 10 splicing has been implicated in a range of sporadic tauopathies, including progressive supranuclear palsy (PSP), corticobasal degeneration (CBD), multiple system tauopathy with dementia (MSTD), and argyrophilic grain disease (AGD), as well as some forms of Alzheimer’s disease (Andreadis, 2006). Our data showing an increase in RBM25 and DDX5 expression in the cerebellum in response to Mn deficiency therefore suggests an involvement of the spliceosome in the Mn-dependent function of the nervous system.

The spliceosome is found within the nucleus in eukaryotic cells. Studies have also shown that Mn is primarily accumulated in the nuclei of several types of cultured brain cells, including blood–brain barrier endothelial RBE4 cells, blood–cerebrospinal fluid barrier choroidal epithelial Z310 cells, mesencephalic dopaminergic neuronal N27 cells, and pheochromocytoma dopaminergic PC12 cells (Kalia et al., 2008). This localization of Mn suggests that these nuclei may serve as the primary pools of intracellular Mn. Currently, the specific roles of Mn in the nucleus are not known. Given the ability of Mn to interact with the nucleotides of DNA, RNA, and ribosomes (Jouve et al., 1975; Vogtherr and Limmer, 1998), one plausible explanation is that unidentified nuclear proteins are specifically targeted by Mn. Further studies are warranted to test this intriguing possibility.

The present results show that Mn deficiency decreases the expression of genes involved in multiple reactome pathways, including carbohydrate metabolism, in the cerebellum. This is consistent with previous findings that Mn deficiency in mice impairs the metabolism of carbohydrates (Wedler, 1993; Keen et al., 1999). Mn acts as a cofactor for numerous enzymes, including all six major enzyme families, and several Mn metalloenzymes contain tightly bound Mn ions (Wedler, 1993). Mn metalloenzymes include pyruvate carboxylase, which catalyzes the physiologically irreversible carboxylation of pyruvate to form oxaloacetate in the citric acid cycle (Leach and Lilburn, 1978). Glycosyltransferase is another Mn-dependent enzyme that requires Mn for catalytic activity (Leach, 1971), implicating Mn as a significant factor in glycosylation. Mn-deficient animals exhibit skeletal abnormalities, including shortened limbs caused by a diminished production of the *N*-acetylgalactosamine-containing chondroitin sulfate (Leach, 1971). β-1,4-galactosyltransferase, which catalyzes the transfer of galactose to the glycan moiety of a protein during protein *N*-glycosylation, is another Mn-dependent glycosyltransferase that requires Mn for substrate binding and catalytic activity (Ramakrishnan et al., 2006). Mn-deficient cells have shown impaired protein N-glycosylation, and especially galactosylation (Potelle et al., 2016). Taken together, our results suggest that the altered carbohydrate metabolism reported in the Mn-deficient condition is likely involved in transcriptional alterations.

Previous transcriptome analyses related to Mn have revealed that high levels of Mn alter gene expression in different cell stress pathways and that these alterations may contribute to the toxic effects of Mn. One transcriptome analysis study in *Caenorhabditis elegans* showed that acute Mn exposure increases activity of pathways related to endoplasmic reticulum and lipocalin (Rudgalvyte et al., 2016). Another transcriptome analysis study in the SH-SY5Y human neuroblastoma cell line showed different responses to physiologic versus toxic Mn levels (Fernandes et al., 2019). This study indicated that exposure to physiologic Mn levels increased the abundance of differentially expressed genes (DEGs) in the protein secretion pathway, which functions in protein trafficking and cellular homeostasis. By contrast, exposure to toxic Mn levels increased the abundance of DEGs for the mitochondrial oxidative phosphorylation pathway. Our transcriptomic analysis in the mouse cerebellum revealed vastly different sets of DEGs when compared to these previous studies. Specifically, low and high Mn levels did not result in opposite changes in gene expression. These somewhat inconsistent observations may be reflect the use of different experimental systems. Alternatively, low or high Mn exposures have distinct biological consequences. Further studies are required to compare exposures to low or high Mn levels in a single experimental system.

One important point to note is that dietary Mn deficiency is rare in humans due to the broad Mn availability in diverse dietary sources; therefore, the argument can be made that our transcriptome study may not accurately apply to humans. Mn is certainly abundant in plant-based foods, such as whole grains, legumes, rice, nuts, and vegetables; however, it is deficient in animal sources, including meat, fish, poultry, eggs, and dairy products (Freeland-Graves et al., 2016). Interestingly, epidemiological surveys have revealed a > 40% reduction in dietary Mn consumption in the past 5 years in the United States (Hallfrisch et al., 1987; Egan et al., 2002; Freeland-Graves et al., 2016), and a similar substantial decline in Mn consumption in China (Association, 2001; Jiang et al., 2015) and in South Korea (Kim et al., 2008; Kang et al., 2014). These studies suggest that, despite the supposed rarity of dietary Mn deficiency, Mn inadequacy is actually prevalent in the animal-based diets of today’s society. Furthermore, this Mn inadequacy coincides with an increased incidence of neurodevelopmental conditions associated with low levels of Mn (Claus Henn et al., 2010; Bhang et al., 2013; Haynes et al., 2015). In contrast to the > 40% chronic reduction of Mn in humans, in our study, mice fed with a Mn-deficient diet for 2 weeks showed a moderate reduction of Mn in the cortex (∼13.1%) and cerebellum (∼14.7%). We speculate that the cell-type composition may influence the sensitivity to dietary Mn levels. This is an important future direction of investigation. Further studies should investigate the consequences of longer durations of Mn deficiency on other brain regions.

Importantly, the Mn reduction tested in the present study was sufficient to cause detectable changes in gene expression. The findings presented here will aid in the assessment and management of the risks of low Mn levels in association with human children’s neurodevelopmental conditions. Our present study will also provide a foundation for this type of research. In addition, our study used only a single omics technology, so our results do not reflect proteomics, metabolomics, or functional outcomes. Further integrated omics and time-course studies in animal models will be needed to improve our understanding of the physiological mechanisms that control Mn availability and to establish the pathogenic mechanisms by which low Mn levels cause neuropathology.

## Conclusion

To our knowledge, this is the first global transcriptomic study to analyze the impact of dietary Mn deficiency on the mouse brain. Our study highlights the transcriptional changes occurring in the spliceosome pathway and in carbohydrate metabolism and supports the current knowledge on the roles played by these pathways in the molecular dysfunction associated with low Mn levels. Our study also can serve as a unique data resource for investigating Mn-dependent cellular functions in the brain.

## Data Availability Statement

The datasets generated in this study can be found in online repositories. The names of the repository/repositories and accession number(s) can be found in the article/Supplementary Material.

## Ethics Statement

The animal study was reviewed and approved by the University Committee on Use and Care of Animals (UCUCA) at the University of Michigan.

## Author Contributions

YS designed the research and wrote the manuscript, provided essential materials, and had primary responsibility for final content. YS, E-KC, LA, MP, and SI conducted the research. YS and SI analyzed data and performed statistical analysis. All authors have read and approved the final manuscript.

## Conflict of Interest

The authors declare that the research was conducted in the absence of any commercial or financial relationships that could be construed as a potential conflict of interest.
