# Supplementary material for: Transcriptome Analysis of the Cerebellum of Mice Fed a Manganese-Deficient Diet
Source: Front Genet. 2020 Dec 3;11:558725. doi: 10.3389/fgene.2020.558725 (PMC7780674; doi:10.3389/fgene.2020.558725)
Supplement: Supplementary file 2 [file Table_1.DOCX]

**Supplemental Table 1** The composition of normal-Mn diet (TD.120518, Teklad)

| **Dietary Components** | g/Kg |
| --- | --- |
| Casein, low Cu & Fe | 200.0 |
| DL-Methionine | 3.0 |
| Sucrose | 549.3452 |
| Corn Starch | 150.0 |
| Corn Oil | 50.0 |
| Vitamin Mix, AIN-76A (40077) | 14.0 |
| Choline Bitartrate | 2.8 |
| Ethoxyquin, antioxidant | 0.01 |
| Calcium Phosphate, dibasic | 15.5 |
| Sodium Chloride | 2.59 |
| Potassium Citrate, monohydrate | 7.7 |
| Potassium Sulfate | 1.82 |
| Magnesium Oxide | 0.84 |
| Zinc Carbonate | 0.056 |
| Cupric Carbonate | 0.0105 |
| Potassium Iodate | 0.0004 |
| Sodium Selenite, pentahydrate | 0.0004 |
| Chromium Potassium Sulfate, dodecahydrate | 0.0193 |
| Manganous Carbonate | 0.0732^1^ |
| Ferric Citrate | 0.235 |

^1^Manganese content approximately 35 - 35.5 mg/kg
