# Supplementary material for: Transcriptome Analysis of the Cerebellum of Mice Fed a Manganese-Deficient Diet
Source: Front Genet. 2020 Dec 3;11:558725. doi: 10.3389/fgene.2020.558725 (PMC7780674; doi:10.3389/fgene.2020.558725)
Supplement: Supplementary file 3 [file Table_2.DOCX]

**Supplemental Table 2** Primer Sequence

| *Ki* | TGA CCC GAA TGT CTA TCT GTG GGA |
| --- | --- |
|  | GCA CGA TAG GTC ATG TTC CGT GTG |
| *Fn1* | GGC CAC CAT TAC TGG TCT GG |
|  | GGA AGG GTA ACC AGT TGG GG |
| *Meg3* | CAG AGC GCT TCT GAA GAC CA |
|  | CAC CTA CTG GGT GCT CAC TG |
| *Tob2* | GTG CAG GTC GTT GAG ACT GA |
|  | ATC CAG AGG CTC TCG GGT C |
| *Blc6* | GAC GTT GTC ATC GTG GTG AG |
|  | GGT TGC ATT TCA ACT GGT CA |
